# Imaging the effects of treatment with TERT and EGFR inhibitors on glioblastoma: An MR study

**DOI:** 10.1093/noajnl/vdaf078

**Published:** 2025-04-17

**Authors:** Donghyun Hong, Noriaki Minami, Sabrina M Ronen

**Affiliations:** Department of Radiology and Biomedical Imaging, University of California, San Francisco, San Francisco, California, USA; Department of Radiology and Biomedical Imaging, University of California, San Francisco, San Francisco, California, USA; Department of Radiology and Biomedical Imaging, University of California, San Francisco, San Francisco, California, USA

**Keywords:** ^1^H MRS, EGFR, glioblastoma, hyperpolarized ^13^C MRS, TERT

## Abstract

**Background:**

Telomerase reverse transcriptase (TERT) promoter mutations are observed in most glioblastoma (GBM) tumors, leading to TERT expression, which is crucial for tumor growth. Accordingly, inhibiting TERT or its upstream tumor-specific transcription factor GA-binding protein transcription factor subunit beta 1 (GABPB1) was shown to inhibit tumor growth. In addition, epidermal growth factor receptor (EGFR) was shown to signal upstream of TERT and GABPB1 and to control TERT expression, and EGFR inhibition also inhibits GBM growth.

**Methods:**

This study investigated the individual as well as combined effects of EGFR, TERT, and GABPB1 inhibition on cell and orthotopic rat models. We assessed cell proliferation, animal survival, tumor size, ^1^H magnetic resonance spectroscopy (MRS)-detectable steady-state lactate, and ^13^C MRS-detectable hyperpolarized (HP) lactate production.

**Results:**

When TERT or GABPB1 were inhibited simultaneously with EGFR, the combination treatment resulted in enhanced inhibition of cell and tumor growth as well as animal survival compared not only to controls but also to any of the single treatments. Our study also found that steady-state ^1^H MRS-detectable lactate and HP ^13^C MRS-detectable lactate production dropped following every treatment, and the drop was significantly greater following combination treatments. Furthermore, the metabolic changes occurred prior to changes in tumor size, and a reversal of these metabolic biomarkers was associated with tumor recurrence.

**Conclusion:**

Our study points to the value of steady-state ^1^H MRS-detectable lactate and HP ^13^C MRS-detectable lactate as potential biomarkers of response to combination EGFR/TERT inhibition.

**Key Points**

- ^1^H MRS-detectable lactate drops significantly following inhibition of EGFR and TERT.
- ^13^C MRS-detectable lactate production also drops significantly.
- The metabolic changes precede changes in tumor size and predict survival.

**Importance of the Study**

This study investigated the effects of treatment with an epidermal growth factor receptor (EGFR) inhibitor and its combination with downstream silencing of telomerase reverse transcriptase (TERT) or the tumor-specific GA-binding protein transcription factor subunit beta 1 (GABPB1) in glioblastoma cell and animal models, and used proton spectroscopy and hyperpolarized (HP) ^13^C magnetic resonance spectroscopic imaging of lactate to identify imaging biomarkers associated with response to treatment. When TERT or GABPB1 were inhibited simultaneously with EGFR, the combination treatment enhanced the inhibition of both cell and tumor growth, resulting in extended animal survival when compared not only to controls but also to using any treatment alone. Our study also found that steady-state ^1^H MRS-detectable lactate and HP ^13^C MRS-detectable lactate production predict the enhanced response to this new combination treatment. Additionally, reversal in these MRS biomarkers was associated with tumor recurrence.

Glioblastoma multiforme (GBM) is a highly aggressive malignant brain tumor primarily affecting adults. There are over 10 000 new GBM diagnoses in the United States each year, and, unfortunately, the overall median survival rate is very low at only 15 months.^1^ Currently, temozolomide (TMZ) is used as a first-line treatment for GBM. This oral alkylating agent induces DNA damage that results in apoptosis and reduces the expression of vascular endothelial growth factor (VEGF) and matrix metalloproteinase (MMP).^1,2^ However, the effectiveness of TMZ is limited by its toxicity^3^ and the fact that GBM tumors develop resistance within 7-10 months of treatment.^4^

Recently, telomerase reverse transcriptase (TERT) has emerged as a novel potential therapeutic target for treating GBM.^5^ TERT expression is the most common mechanism by which cancers maintain telomere length and achieve immortality, and up-regulation of TERT expression due to TERT promoter mutations is a prominent genetic feature in GBM. TERT mutations are observed in 80% of wild-type isocitrate dehydrogenase (IDH) GBM and are associated with poor prognosis.^6^ Furthermore, TERT promoter mutations can induce high transcriptional activity under hypoxic and TMZ-treated conditions.^7^ In contrast, when TERT is inactivated, GBM cells show a shortening of telomeres and the formation of chromatin bridges, losing their ability to proliferate.^8^

Previous studies^7,9^ have shown that TERT promoter mutations are novel prognostic markers for glioma and can inform potential treatment strategies. However, currently available telomerase inhibitors have several limitations. The anti-telomerase compound Imetelstat or GRN163L, which has been used clinically, was not very effective, especially in solid tumors, and also caused significant hematologic toxicity.^10^ Moreover, inhibiting TERT is also detrimental to normal stem and germline cells, leading to challenges for telomerase-targeted therapy in the clinic.^11^ Importantly, however, recent studies have shown that GA-binding protein transcription factor subunit beta 1 (GABPB1) is a tumor-specific TERT transcription factor. This identifies a tumor-specific approach to control TERT expression and thus inhibit tumor growth with limited toxicity. Another slight limitation of existing TERT inhibitors is that they require multiple cell divisions before cell death.^8^ However, epidermal growth factor receptor (EGFR) amplification and TERT promoter mutations commonly occur together in GBM,^12^ and recent studies have shown that EGFR signaling can further regulate TERT expression by regulating the expression of GABP.^12^ These studies have also shown that targeting the EGFR pathway has the potential to enhance the effectiveness of TERT inhibition. A different study also showed the potential of combination treatment targeting EGFR in recurrent GBM,^13^ and Gefitinib, which is a first-generation reversible ATP-site competitive EGFR kinase inhibitor, was shown to be effective in treating GBM as well as other cancers.^14,15^

Response to therapy is typically assessed by using imaging to determine tumor size, and in the clinical setting, this is achieved for most GBM cases by using magnetic resonance imaging (MRI).^16^ However, MRI does not provide predictive information, and anatomic information can be ambiguous if tumor stasis or inhibition of tumor growth, rather than shrinkage, is observed. In contrast, magnetic resonance spectroscopy (MRS) has been shown to provide information regarding tumor grade and predict response to treatment.^17–19^ MRS is a noninvasive, radiation-free method that can detect metabolic information from cells, tissues, animal models, and patients. Proton (^1^H) MRS detects the steady-state levels of endogenous metabolite, and hyperpolarized (HP) ^13^C MRS provides an in vivo assessment of enzymatic fluxes.^20^ Our recent studies in GBM have shown that ^1^H MRS-detectable lactate levels and HP ^13^C MRS-detectable levels of hyperpolarized lactate production are associated with the modulation of TERT expression either directly via targeting of TERT or upstream via targeting at the level of GABPB1 in GBM cell and animal models.^21,22^ Earlier studies from our lab and others have also shown that inhibition of EGFR or some of its downstream signaling pathways is also associated with changes in hyperpolarized lactate production.^23,24^

The goal of the current study was, therefore, to investigate the impact of dual EGFR and TERT/GABPB1 inhibition, confirm the efficacy of this combination treatment, and determine whether proton MRS-detectable lactate combined with HP lactate can provide early indicators of response and potential drug resistance. To that end, we performed a small-scale cell study and then expanded our investigations to tumor-bearing animals.

## Methods

### Cell Models

We utilized our previously described cell models^21^: U251shCtrl, U251shTERT, U251shGABPB1 (U251shB1), GS2shCtrl, GS2shTERT, and GS2shGABPB1 (GS2shB1). Briefly, the well-established U251 GBM model was obtained from the American Type Culture Collection. The GS2 patient-derived GBM model was obtained from the UCSF Brain Tumor Center Preclinical Therapeutics Core. TERT or GABPB1 expression was silenced in those cells using shRNA transduction. Cells were also transduced with control shRNA to serve as control cells.^21^ All cells were maintained in a 37°C incubator with 5% CO_2_. U251-derived cells were cultured in DMEM/Ham’s F-12 supplemented with 10% fetal bovine serum (FBS), 2 mM glutamine, 100 U/mL penicillin, and streptomycin. GS2-derived cells were cultured in DMEM supplemented with 10% FBS, 2 mM glutamine, 100 U/mL penicillin, and streptomycin. All cell lines were routinely tested for mycoplasma and authenticated by short tandem repeat fingerprinting within 6 months of any experiment.

### Animal Models

All animal studies were approved by the UCSF Institutional Animal Care and Use Committee. Orthotopic tumors were induced in nude rats (RH-Foxn1rnu, 150-200 g, and 6-7 weeks, Envigo IN) as previously^21^ using control, TERT-silenced, and GABPB1-silenced U251 or GS2 GBM cell lines (U251shCtrl, U251shTERT, U251shB1, GS2shCtrl, GS2shTERT, and GS2shB1). Tumor growth was monitored periodically by T_2_-weighted MR imaging on a preclinical 3 T scanner (Biospec, Bruker) equipped with a dual-tuned ^1^H-^13^C volume coil (40-mm inner diameter, Bruker). When tumors were large enough to perform MR spectroscopy (approximately 27 mm^3^), we considered this as the study starting point (Day 0), and animals were treated as described below with vehicle or the EGFR inhibitor Gefitinib daily until the endpoint. Tumor growth was then monitored as above by MRI every 7 days, and after 10 days of treatment, the ^1^H and HP ^13^C MRS studies acquired the posttreatment data set (*n* = 6 for each group). Animals were treated, and their tumor growth and survival rates were monitored until the endpoint. When possible, we also acquired ^1^H and HP ^13^C MRS data immediately before the animal’s endpoint.

### Treatment

Cells and animals were treated with the EGFR inhibitor Gefitinib (#184475-35-2, Fisher Scientific). For cells, U251shCtrl, U251shTERT, U251shB1, GS2shCtrl, GS2shTERT, and GS2shB1 cells were treated every 24 h for 3 days with the half-maximal inhibitory concentration (IC_50_) of Gefitinib (52 µM)^25^ or DMSO (vehicle, 0.2%, MilliporeSigma) (*n* = 3 in each group). For animal studies, animals were injected intraperitoneally with a concentration of 80 mg/kg body weight of Gefitinib^26^ every 24 h until their endpoint. Fifty microliters of DMSO plus 3 mL of saline was used as a vehicle. Control animals were treated with vehicle only.

### Cell MRS Studies

Approximately 5 × 10^7^ cells were extracted using dual-phase extraction^27^ (*n* = 3 for each group). The lyophilized aqueous phase was resuspended in 400 µL D_2_O-based phosphate buffer with sodium 3-trimethylsilyl propionate-2,2,3,3-d4 (TSP) as an external reference. Proton spectra were acquired using a 90-degree pulse on an MR spectrometer (500 MHz, Bruker). Spectra were analyzed using MNOVA (Mestrelab Research). Metabolites were quantified by integration, corrected for saturation, and normalized to cell number and TSP.^28^

### In Vivo Proton Single Voxel MRS

Proton single voxel spectroscopy was acquired from a 4 × 4 × 4 mm^3^ voxel within the tumor region of each animal using the PRESS sequence with short TE = 16 ms (TE1 = TE2 = 8 ms), TR = 2500 ms, averages = 512, 1024 data points, spectral bandwidth = 10 ppm, and using the VAPOR water suppression.^29^ One-Hertz line broadening was applied to remove high-frequency noise. LCModel^30^ was used to quantify the spectra using an absolute quantification approach using the unsuppressed water signal.^31^ The basis set for LCModel fitting included 19 metabolites^21^ and was created using the NMRSIM package in the Topspin suite (Bruker) with identical parameters used for the in vivo acquisition and with chemical shift and J-coupling values from a previous study.^32^ In order to further quantify lactate without the interference of the lipid signal at 1.3 ppm, we also acquired a long TE (TE = 144, TE1 = 30 ms, and TE2 = 114 ms) PRESS data set from some of the U251 animals. Identical quantification methods were used as for the short TE data, but with a corresponding long TE basis set.^33^ The basis set took into account the potential signal intensity differences due to TE differences. To evaluate the quality of the acquired spectra, the signal-to-noise ratio (SNR) was calculated from a non-apodized spectrum by using the maximum height of the largest signal divided by the standard deviation of the noise.^34,35^

### In Vivo Hyperpolarized ^13^C MRSI

Twenty-four microliters of [1-^13^C]pyruvate was hyperpolarized using a Hypersense polarizer (Oxford Instruments), dissolved in 4.5 mL of 80-mM buffer containing 80 mM NaOH, 40 mM Tris, and 0.3 mM EDTA for dissolution and pH balance, and injected via the tail vein over 10 s.^21^ Spectra were acquired using a flyback spectral-spatial echo-planar spectroscopic imaging (EPSI)^36^ sequence with flip-angle = 30° for [1-^13^C]lactate and 6° for [1-^13^C]pyruvate. The RF pulse was designed using the in-house software package available online (https://github.com/LarsonLab/hyperpolarized-mri-toolbox) in Matlab (ver. 2024a, MathWorks). Spatial resolution = 2.5 × 2.5 × 8 mm^3^, temporal resolution = 3 s, 20 repetitions, 256 data points. Spectral signal to noise was improved using Tensor denoising.^37^ Spectra were processed using a custom-written Matlab script (https://github.com/donghyunh/2DEPSI_Analysis.git). We then quantified lactate and pyruvate in each tumor voxel and each normal contralateral brain voxel in each animal. To avoid any partial volume effects, we then considered only data from voxels that were either entirely within the tumor or entirely within the normal contralateral brain and averaged this data (3-4 voxels) in order to remove any potential bias in data selection. Metabolites were quantified using each metabolite’s area under the curve (AUC). The AUC of the lactate and pyruvate peaks was quantified at each time point and normalized to the maximum pyruvate peak to monitor metabolism over time. Dynamic HP lactate heatmaps were generated based on the normalized lactate AUC from the EPSI spectra of each voxel at each time point. The temporal evolution of the HP lactate signal was quantified based on the normalized AUC of the lactate peak from each voxel over time, and total lactate production was determined from the sum of these AUCs.

### Statistics

For cell studies, all experiments were repeated 3 times (*n* = 3). For animal studies, all experiments were performed 6 times (*n* = 6) except the long TE proton SVS acquisition, for which *n* = 3, and endpoint data, which was acquired from *n* = 4 in the U251 model and from *n* = 3 for the GS2 model (reduced endpoint numbers were due to animal death). Results were represented in mean ± SD. For cell studies, an unpaired Student’s *t* test was used to compare control and treated groups, assuming unequal variance for 2-group comparisons. For animal studies, survival was assessed using Kaplan-Meier survival curves with the Log-rank test to assess significance. Metabolites were compared using a 1-way analysis of variance (ANOVA) with Tukey’s post hoc test to compare the metabolite concentrations in each of the 6 groups. PRISM 10 (GraphPad Software Inc.) was used to perform the statistical analyses. A *P*-value ≤ .05 was considered statistically significant. * Signifies *P*-value < .05, **P-value < .01, ***P-value < .001, and ns = not significant.

## Results

### Cell Results

**Figure 1.**
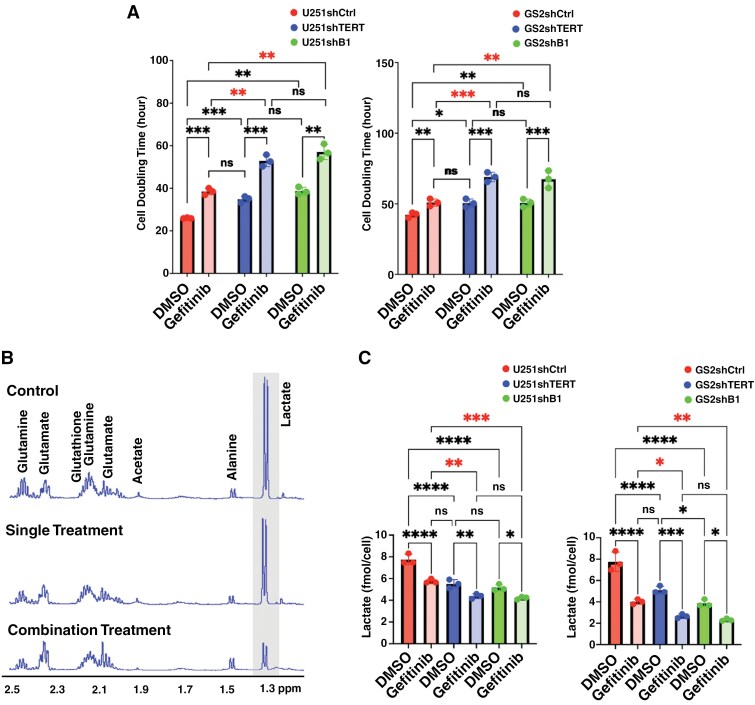
(A) Cell doubling time in U251 (left) and GS2 (right) models. (B) Representative ^1^H NMR spectra from U251 control, single-treated (TERT), and dual-treated (TERT + EGFR) cells, respectively. (C) Lactate quantification based on ^1^H MRS spectra from U251 (left) and GS2 (right) cell lines. *P-value < .05, **P-value < .01, ***P-value < .001, and ns = not significant. The combination effects are highlighted with a red asterisk.

First, we investigated the impact of TERT, GABPB1, and EGFR inhibition on cell proliferation (Figure 1A). Consistent with our previous results,^21^ when TERT or GABPB1 were inhibited, the cell doubling time significantly increased in both our cell models (U251 and GS2) compared to controls (increased by 19.6% for TERT inhibition, and 19.8% for GABPB1 inhibition in U251; 34.3% for TERT inhibition, and 49.1% in GS2). Similarly, when EGFR was inhibited, the cell doubling time increased (by 20.6% for U251 and 48.3% for GS2). Interestingly, the effect of EGFR inhibition was comparable to that of TERT or GABPB1 silencing alone. Importantly, when both TERT and EGFR, or GABPB1 and EGFR were simultaneously inhibited, cell growth was significantly inhibited beyond the inhibition observed with a single treatment in both cell models (increased cell doubling time by 63.4% from control for TERT and EGFR inhibition, and 59.8% for GABPB1 and EGFR inhibition in U251; 103.8% for TERT inhibition and EGFR inhibition, and 120.1% for GABPB1 and EGFR inhibition in GS2).

Using the ^1^H MRS data of the cells (Figure 1B), we compared their steady-state intracellular metabolites. We focused on lactate, which we had previously found to be a biomarker of response to TERT silencing.^21^ We found that, consistent with our previous results,^21^ lactate levels dropped significantly (by 28.9% from control for TERT inhibition and 33.4% for GABPB1 inhibition in U251, 34.1% for TERT inhibition, and 50.1% for GABPB1 in GS2) when TERT or GABPB1 were silenced (see Figure 1C). Lactate also decreased significantly following EGFR inhibition (28.9% for U251 and 34.1% for GS2). Moreover, the combination treatments in both cell lines led to even lower lactate levels compared to the single treatments (red asterisk in Figure 1C) (dropped by 43.5% for TERT and EGFR inhibition and 44.4% for GABPB1 and EGFR inhibition in U251; 65.7% for TERT and EGFR inhibition and 70.1% for GABPB1 and EGFR inhibition in GS2).

### In Vivo Animal Results

**Figure 2.**
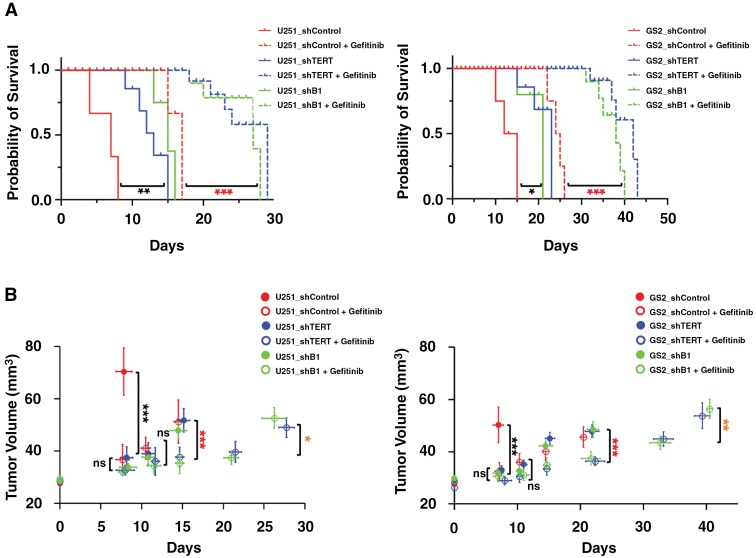
(A) Kaplan-Meier survival plot of U25 (left) and GS2 (right) tumor-bearing rats. (B) Temporal evolution of average tumor volume in mm^3^ for U251 (left) and GS2 (right) animals based on T2-weighted MR images. *P-value < .05, **P-value < .01, ***P-value < .001, and ns = not significant.

Figure 2A shows a significant increase in survival across all treatment groups compared to the control group. Specifically, in the U251 model, EGFR inhibition increased survival by 130%, while TERT and GABPB1 inhibition led to 104% and 118% increases, respectively. In the GS2 model, survival increased by 92% with EGFR inhibition, 44% with TERT inhibition, and 37% with GABPB1 inhibition. Furthermore, consistent with the cell studies, the combination treatment group survived significantly longer than the single treatment groups for both tumor models (increased by 291% from the control group for EGFR and TERT inhibition and 293% for EGFR and GABPB1 inhibition for U251, 200% for EGFR and TERT inhibition, and 191% for EGFR and GABPB1 inhibition for GS2).

Figure 2B illustrates tumor growth as measured using T_2_-weighted MR images for U251 (left) and GS2 (right) animals. We observed a similar trend in tumor growth for both tumor models. The control group animals that did not receive any treatment showed rapid growth, but tumor growth was inhibited when we inhibited a specific target. Tumor volume around Day 8 was 70.4 ± 7.8 mm^3^ for control and 36.0 ± 8.1 mm^3^ for single-treated U251 animals; 50.3 ± 6.2 mm^3^ for control and 32.5 ± 7.2 mm^3^ for single-treated GS2 animals; *P*-value < .001 for both models. Furthermore, the combination treatments (EGFR and TERT or EGFR and GABPB1) led to even greater tumor growth inhibition than the single treatments. The difference in tumor growth between single-treated and dual-treated was significant on Day 15 for U251 (50.3 ± 14.7 mm^3^ for single-treated and 36.6 ± 5.8 mm^3^ for dual-treated) and on Day 22 for GS2 tumors (42.5 ± 10.3 mm^3^ for single-treated and 34.2 ± 7.1 mm^3^ for dual-treated).

**Figure 3.**
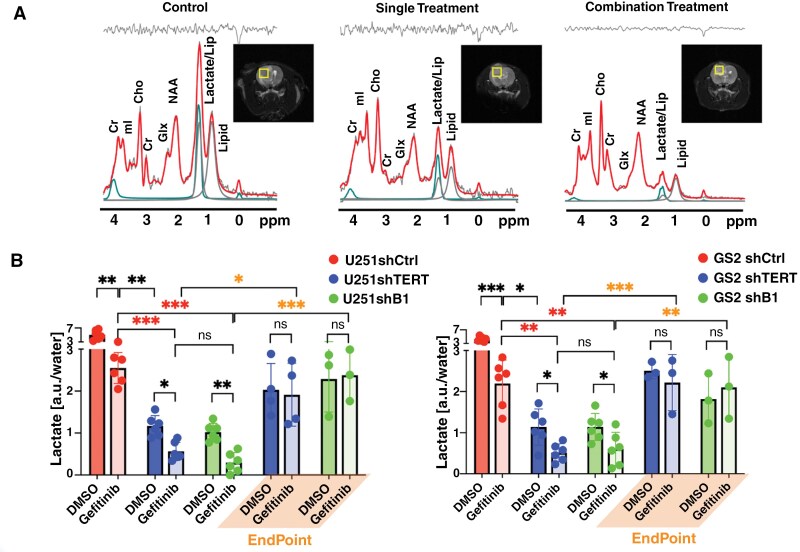
(A) Representative in vivo ^1^H MRS spectra with LCModel quantification. Image inserts illustrate the location of 4 × 4 × 4 mm^3^ voxel inside the tumor from which spectra were acquired from U251 control, U251 single-treated (shTERT), and U251 dual-treated (shTERT + Gefitinib) tumors. The in vivo spectra are illustrated in light gray, and the overlapping red lines indicate the LCModel fitting line. The fit to lipid resonances at 0.9 ppm (dark blue) and 1.3 ppm (light blue) is illustrated, and the fit to lactate at 1.3 and 4.09 ppm is illustrated in green. The upper panel shows the residual signal. (B) Quantified lactate levels of U251 (left) and GS2 (right) animals at 10 days of treatment and endpoint. The significance of combination treatment effects is highlighted with red asterisks, and orange asterisks highlight the significance of observations at the endpoint. *P-value < .05, **P-value < .01, ***P-value < .001, and ns = not significant.

Figure 3A illustrates proton spectroscopy data with the corresponding voxel locations and LCModel fitting lines. It should be noted that LCModel fits lactate at 1.33 ppm and 4.0974 ppm and lipids at 1.3 ppm and 0.9 ppm, which helps address the proximity of the lipid and lactate peaks around 1.3 ppm. Given the limited sensitivity of MRS, we cannot detect every single metabolite within the tumor. However, the LCModel analysis indicated that of the 15 metabolites that are detectable by MRS in our study, lactate was the only metabolite consistently modulated by treatment in both models (see Supplementary Table S1). Figure 3B illustrates lactate quantification. Control animals showed a relatively high lactate level (5.0 ± 1.4 AU for U251 and 4.2 ± 0.7 AU for GS2). When either EGFR, TERT, or GABPB1 were inhibited, lactate levels were lower than in controls and, in contrast to the cell data, TERT or GABPB1-silenced tumors showed a greater reduction in lactate levels than EGFR-treated tumors (lactate dropped by 49.4%, 76.8%, and 79.7% for U251; 47.7%, 72.9%, and 68.3% for GS2; EGFR inhibited, TERT inhibited, and GABPB1 inhibited, respectively). Importantly, however, and consistent with the cell data, when both TERT or GABPB1 were inhibited simultaneously with EGFR, lactate levels dropped to a greater extent than with any of the single treatments (dropped by 88.8% and 94.1% compared to the control group for U251; 87.7% and 85.6% for GS2; EGFR/TERT inhibition and EGFR/GABPB1 inhibition, respectively). Furthermore, the drop in lactate was significant at 10 days, a time point at which tumor size alone would not be able to predict treatment response and enhanced animal survival. In addition, when these animals approached their endpoints, lactate levels increased again, even though the treatments were continued. These trends were the same in both tumor models, although lactate levels were generally lower in the GS2 model. At the endpoints, lactate levels in U251 tumors returned to 57.1% of the control group for single treatments and 55.4% for dual treatments. In GS2 tumors, 48.5% for single treatments and 45.4% for dual treatments.

We also conducted a small-scale study (*n* = 3) with optimized acquisition conditions to further confirm our findings and measure lactate without interference from the lipid peak at 1.3 ppm using a long TE PRESS sequence. Supplementary Figure S1 shows a similar trend in lactate levels compared to the short TE measurement (dropped by 37.7%, 64.7%, and 75.1% compared to control for U251, respectively). This confirmed that the lactate signal is the primary contributor to the 1.3 ppm peak in our models, and its modulation is associated with treatment effects.

Supplementary Table S2 illustrates the Cramér-Rao lower bound (CRLB) values used to evaluate the spectral fitting quality of the LCModel. LCModel quantification with CRLB of below 50% is considered reliable.^38^ Here, our CRLBs for lactate were below 11% for short TE data and below 26% for long TE data. These values indicated that our SNR (46-94 for short TE data and 28-40 for long TE data, see Supplementary Table S3) and spectral fitting quality were acceptable to quantify lactate precisely.

**Figure 4.**
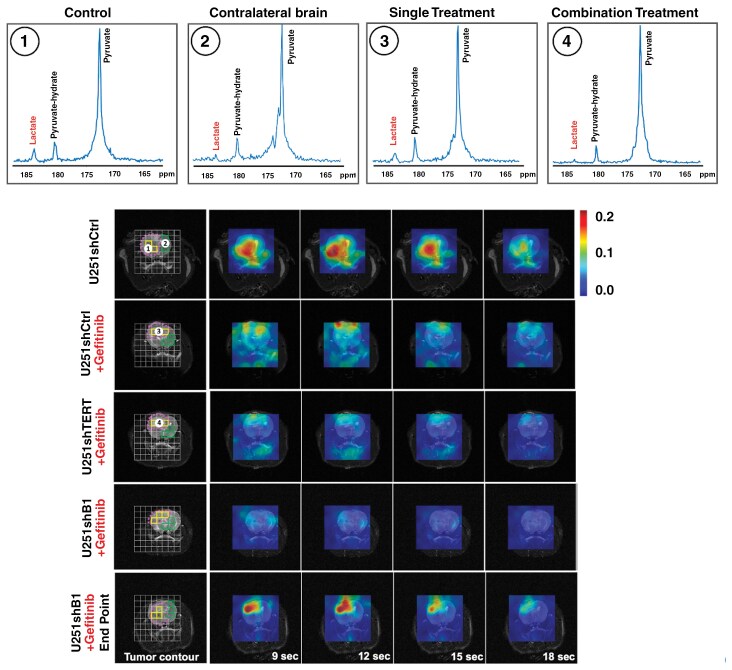
Dynamic heat maps of hyperpolarized [1-^13^C]lactate were acquired using an EPSI sequence from each tumor model with a spatial resolution of 2.5 × 2.5 × 8 mm^3^ and a temporal resolution of 3 s. The lactate signal was normalized to the maximum pyruvate signal. Highlighted voxels in yellow (tumor) and green (contralateral) indicate the voxels used for the quantification. The upper panel shows representative summed ^13^C spectra over the first 10 time points after injection of hyperpolarized [1-^13^C]pyruvate (from 3 to 30 s) from a representative single voxel of (1) control tumor (U251shControl), (2) contralateral brain (U251shControl), (3) single treatment tumor (U251shTERT), and (4) combination treatment tumor (U251shTERT plus Gefitinib).

Lactate is known to accumulate in necrotic regions in GBM.^39^ As a result, the proton MRS signal is not necessarily a specific readout of metabolic events occurring in live cells. In contrast, HP ^13^C MRS provides a rapid readout of metabolism that occurs specifically in live cells. Next, we investigated the dynamic production of lactate using HP ^13^C MRS of pyruvate to have a more reliable metric of the metabolic changes occurring in live cells in response to treatment. Representative summed HP spectra over the first 10 time points from a single U251 voxel (2.5 × 2.5 × 8 mm^3^) of the EPSI acquisition matrix are shown in Figure 4A, and a spectrum from the normal contralateral brain. The control tumor spectrum showed the highest lactate level. When TERT was inhibited, the lactate level was reduced. It was further reduced when both EGFR and TERT were inhibited simultaneously, coming down to levels that were comparable to those observed in the contralateral brain. Figure 4B shows representative dynamic HP lactate heatmaps from 9 to 18 s after HP pyruvate injection, generated based on the lactate AUC from the EPSI spectra obtained in the tumor region and contralateral brain. Highlighted voxels in yellow (tumor) and green (contralateral) indicate the voxels used for the quantification. Lactate was highest in the tumor region of the control animals, and lactate levels were reduced with treatment. Interestingly, however, they increased again prior to the animal endpoint.

**Figure 5.**
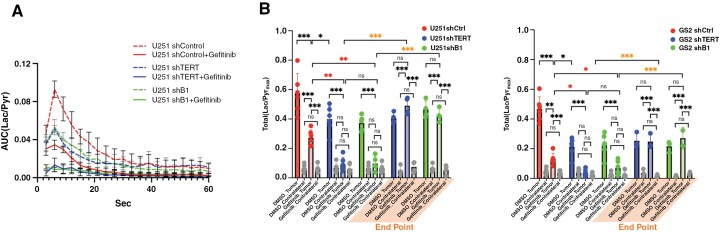
(A) Average dynamic lactate to pyruvate ratio of tumor voxels from each tumor model. (B) Total lactate levels comparing control, single treatment, dual treatment groups, and their corresponding contralateral brains, as well as dual treatment endpoint data for U251 (left) and GS2 (right) animals. The significance of combination treatment effects is highlighted with red asterisks, and the significance of observations at the endpoint is highlighted with orange asterisks. *P-value < .05, **P-value < .01, ***P-value < .001, and ns = not significant.

Figure 5A illustrates the temporal evolution of HP [1-^13^C]lactate averaged across the tumor voxels and all animals from each treatment group. Figure 5B illustrates the average HP lactate production in U251 (left) and GS2 (right) animals within the tumor and the normal contralateral brain. The HP MRS results were consistent with the steady-state proton MRS findings, indicating a clear change in dynamic lactate production. Tumors in which TERT, GABPB1, or EGFR alone were targeted showed lower HP lactate production than the control group (dropped by 52.5%, 30.4%, and 45.7% for U251; 72.3%, 54.6%, and 51.8% for GS2; TERT, GABPB1, or EGFR inhibition, respectively). Furthermore, and importantly, the dual-treated groups showed even lower lactate production than the control group (dropped by 84.1% and 75.4% for U251; 91.9% and 85.2% for GS2; TERT/EGFR inhibition and GABPB1/EGFR inhibition, respectively). These lower lactate levels were within experimental error of the values observed within the contralateral brain. Furthermore, lactate levels in the contralateral brain did not show any statistically significant changes following any of the treatments. Again, as in the case of the ^1^H MRS lactate data, we were able to detect the metabolic changes prior to any change in tumor size. Finally, consistent with our ^1^H data, HP lactate levels prior to the animal endpoints increased again in dual-treated animals, reaching 85.4% and 74.2% of the control levels for U251, and 52.9% and 58.0% for GS2, for TERT/EGFR inhibition and GABPB1/EGFR inhibition, respectively. Our findings were comparable in both our U251 and GS2 models.

## Discussion

GBM is characterized by tumor heterogeneity, extreme clonal plasticity, and the presence of cancer stem cells, invariably resulting in resistance to therapy and postsurgical tumor recurrence.^40^ TMZ is the standard DNA-targeting chemotherapy for GBM treatment, but new therapeutic targets, as well as combination treatments,^41^ are required to enhance patient survival. One potential therapeutic approach is targeting TERT expression, which is essential for tumor growth.^42^ A previous study confirmed that silencing TERT, or its tumor-specific promoter GABPB1, is an effective therapeutic approach.^21^ Another potential therapeutic approach is to target EGFR since EGFR amplifications occur commonly in GBM. TERT promotor mutations typically co-occur with EGFR amplifications,^12,43^ and it has also been shown that EGFR ultimately signals to GABPB1 and TERT. As a result, combining TERT and EGFR inhibition has been proposed as a new combination therapy.^12^ In this study, we, therefore, examined TERT or GABPB1 silencing, combined with inhibition of EGFR signaling in 2 GBM models, and assessed the value of MRS to detect response. We found that tumor growth was inhibited to a greater extent with the dual treatment than with either of the single treatments, and animal survival was also further extended. We also identified proton MRS-based lactate and HP ^13^C MRS-based lactate production as complementary metabolic biomarkers of response, demonstrated that the biomarkers are present early and prior to any change in tumor size and that they are also modulated before animal endpoint, providing possible predictors of response as well as tumor recurrence.

The changes in lactate and lactate production are identical to our previous study,^21^ in which we investigated the silencing of TERT or GABPB1. This is consistent with the fact that EGFR signals to GABPB1 and TERT. It is also consistent with our previous findings that TERT modulates glycolysis.^21^ Importantly, the greater reductions observed with the dual treatment in lactate and HP lactate are consistent with the greater impact of the dual treatment on tumor growth and animal survival. It is, however, important to note that this biomarker is not specific to response to this therapeutic approach. Indeed, it is widely recognized that cancer cells undergo a significant metabolic change compared to normal cells, shifting from oxidative phosphorylation to glycolysis in both hypoxic and normoxic environments due to the Warburg effect.^35,44,45^ This shift leads to the production of substantial amounts of lactate, which, in turn, has been shown to have multiple roles that promote and enhance tumor development. Specifically, exported lactate acidifies the tumor environment, provoking a local inflammatory response that ultimately drives tumor cell growth, invasion, and metastasis. Lactate in the microenvironment also impairs the immune response, disabling immune surveillance. Lactate further promotes tumorigenesis and growth by acting as a signaling molecule to induce endothelial cell migration, tube formation, and tumor angiogenesis. Finally, lactate has also been shown to serve as a biosynthetic carbon source, enabling tumor progression.^46^ Total lactate levels and HP lactate production have, therefore, been identified as valuable biomarkers of the presence of tumors, as well as biomarkers of response following a wide range of treatments in multiple tumor types, including brain tumors.^47,48^

In this study, we did not investigate the specific mechanisms that might explain our findings. Data from the contralateral brain did not show any metabolic changes following treatment, indicating that changes in perfusion or blood-brain barrier permeability are likely not major contributors to our findings, although their role cannot be fully ruled out. With regard to the enzymatic mechanisms underlying our findings, we cannot be certain whether our observations are mediated by changes in monocarboxylate transporter (MCT) expression, lactate dehydrogenase A (LDHA) expression, and/or LDHA activity. However, our previous studies found that TERT inhibition leads to a drop in both MCT and LDHA expression.^21^ Previously, we found that inhibition of signaling downstream of EGFR leads to a decline in LDHA expression and activity.^18,23^ It is, therefore, not unreasonable to assume that changes in MCT and LDHA also explain the observations made in this study.

The ^1^H MRS lactate peak at 1.33 ppm and the lipid peak at 1.3 ppm are not clearly distinguishable in vivo and at the field strength used in this study. As a result, our quantification of lactate relied mainly on the LCModel fitting. To ensure accurate lactate level measurement without interference from the overlapping lipid signals, we also obtained single voxel PRESS spectra with a TE of 144 ms. At this echo time, the lactate methyl doublet was inverted due to scalar (J) coupling,^49^ which validated our LCModel-based quantification. However, the sample size of this study was limited, and although the long echo study only served to confirm our short echo findings, the small sample size is a limitation of this study. Another limitation is the fact that we only acquired the proton data from a single tumor voxel. In the clinical setting, a spectroscopic imaging approach would be more appropriate.

Another potential limitation of our study is that we used shRNA to silence TERT or GABPB1. We did this because existing TERT inhibitors have yet to be very successful in the clinic and are toxic to normal cells. However, a recent paper from Bell et al.^50^ showed that GABPB1 is specific to tumors and activates the mutant TERT promoter in several types of cancer, including GBM. As a result, GABPB1 is now considered a promising target for inhibiting TERT and treating gliomas without toxicity. GABPB1-targeting drugs are not yet available. However, our study confirms their value nonetheless and identifies biomarkers that can help rapid, noninvasive response detection both during drug development and in the clinical setting.

Proton MRS is a noninvasive method that provides quantitative biochemical information regarding steady-state intracellular as well as extracellular metabolites. It is a valuable tool in clinical practice, enhancing diagnostic accuracy, treatment monitoring, and research capabilities.^51^ HP ¹³C MRS enables real-time tracking of metabolic fluxes. Monitoring the fate of pyruvate informs on dynamic lactate production from live cells rather than just steady-state concentrations. Importantly, lactate is known to accumulate in necrotic regions.^39^ Therefore, its steady-state proton signal would include both the static, accumulated, extracellular lactate and lactate that is dynamically being produced by live cells. Because our goal is to monitor the metabolic changes that occur in live cells in response to treatment, HP ^13^C pyruvate MRS provides complementary information to that provided by proton MRS and holds promise for enhancing clinical studies, particularly in heterogenous and necrotic tumors such as GBM. [1-^13^C]pyruvate is the most mature and widely used HP agent and has been shown to have translational value.^52^ When combined, ^1^H and HP ^13^C MRS lend confidence that any metabolic changes observed in lactate following treatment reflect changes within the tumor region and are associated with tumor response. This points to the translational value of our findings for monitoring response to treatment in the clinic.

In summary, our study identified ^1^H MRS-detectable lactate, combined with ^13^C MRS-detectable HP lactate production, as metabolic biomarkers of TERT and EGFR-targeted combination therapy. These imaging biomarkers occurred early and could predict both response and, at a later time point, tumor recurrence. Further studies are necessary to confirm our observations, but given the noninvasive, nondestructive, and translational nature of our biomarkers, our study points to the potential value of MRS in helping monitor response to treatment in patients with GBM.

## Supplementary Material

vdaf078_suppl_Supplementary_Material

## Data Availability

The deidentified data supporting this study’s findings are privately owned and will be made available upon reasonable request, per Neuro-Oncology Advances’ data-sharing policy. Interested researchers may contact the corresponding author to request access to the underlying data used in this study, subject to the institutional review process and data use agreements.
