# Supplementary material for: Imaging the effects of treatment with TERT and EGFR inhibitors on glioblastoma: An MR study
Source: Neurooncol Adv. 2025 Apr 17;7(1):vdaf078. doi: 10.1093/noajnl/vdaf078 (PMC12130967; doi:10.1093/noajnl/vdaf078)
Supplement: vdaf078_suppl_Supplementary_Material [file vdaf078_suppl_supplementary_material.docx]

**Supplementary materials**


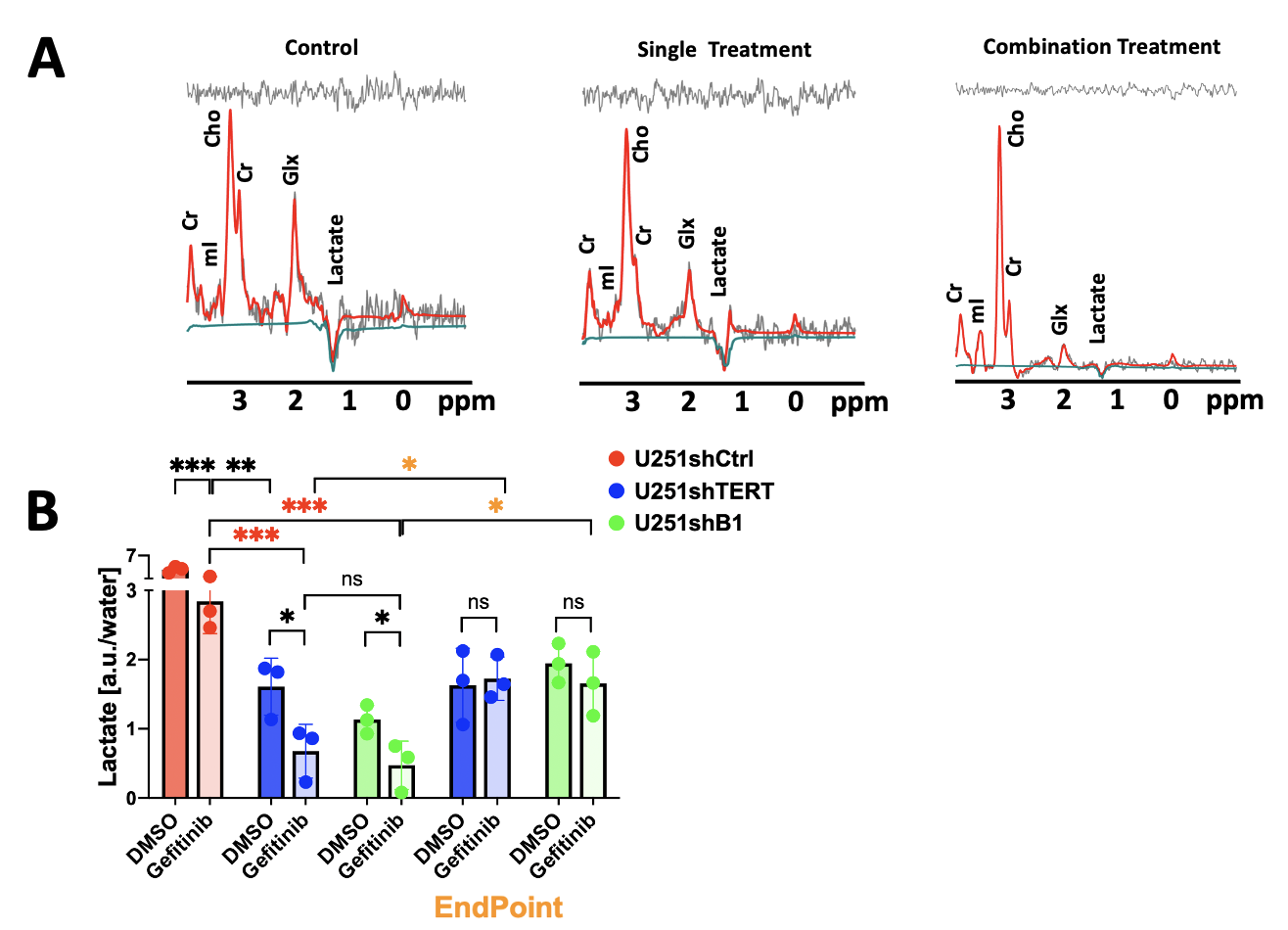


Supplementary Figure S1. (A) Representative in vivo ^1^H MRS spectra acquired with PRESS TE =144 ms from a 4 × 4 × 4 mm^3^ voxel inside the tumor region with lactate levels from LCModel quantification of U251Control, U251shTERT, and U251shTERT plus Gefitinib treatment. The total fitting line is in red, the lactate fitting line is in green, and the upper panel is the residual signal. (B) Quantified lactate levels of U251 animals at 10 days and the endpoint. The significance of combination treatment effects is highlighted with red asterisks, and the significance of observations at the endpoint is highlighted with orange asterisks. * signifies p-value < .05, ** p-value < .01, *** p-value < .001, and ns = not significant.

Supplementary Table S1. Statistical significance comparison between metabolic concentration and groups was estimated by LCModel quantification from proton single voxel spectroscopy for U251(A) and GS2 (B) models. ANOVA with a Tukey’s post hoc test estimated statistical significance. A p-value ≤ .05 was considered statistically significant.

1. U251 Model

|  | **Single**  **treatment effect** | | |  | **Combination**  **treatment effect** | |  |
| --- | --- | --- | --- | --- | --- | --- | --- |
|  | **Control +DMSO**  vs  **Control +Gefitinib** | **shTERT + DMSO**  vs  **shTERT +Gefitinib** | **shB1 + DMSO**  vs  **shB1 +Gefitinib** | **Control +Gefitinib**  vs  **shTERT + DMSO,** | **Control +Gefitinib**  vs  **shTERT +Gefitinib** | **Control +Gefitinib**  vs  **shB1 +Gefitinib** | **shTERT +Gefitinib**  vs  **shB1 +Gefitinib** |
| GSH | ns | ns | ns | ns | ns | ns | ns |
| Lac | *p<0.01* | *p<0.05* | *p<0.01* | *p<0.01* | *p<0.001* | *p<0.001* | ns |
| Glc | ns | ns | ns | ns | ns | ns | ns |
| PE | ns | ns | ns | ns | ns | ns | ns |
| Asc | ns | ns | ns | ns | ns | ns | ns |
| Asp | ns | ns | ns | ns | ns | ns | ns |
| Tau | ns | ns | ns | ns | ns | ns | ns |
| GABA | ns | ns | ns | ns | ns | ns | ns |
| Ala | ns | ns | ns | ns | ns | ns | ns |
| Scyllo | ns | ns | ns | ns | ns | ns | ns |
| Cho+GPC+PCh | ns | *P<0.05* | *p<0.001* | ns | *P<0.05* | ns | ns |
| NAA+NAAG | ns | ns | ns | ns | ns | ns | ns |
| mI+Gly | ns | ns | ns | ns | ns | ns | ns |
| Cr+PCr | ns | ns | ns | ns | ns | ns | ns |
| Glu+Gln | ns | ns | ns | ns | ns | ns | ns |

1. GS2 Model

|  | **Single**  **treatment effect** | | |  | **Combination**  **treatment effect** | |  |
| --- | --- | --- | --- | --- | --- | --- | --- |
|  | **Control +DMSO**  vs  **Control +Gefitinib** | **shTERT + DMSO**  vs  **shTERT +Gefitinib** | **shB1 + DMSO**  vs  **shB1 +Gefitinib** | **Control +Gefitinib**  vs  **shTERT + DMSO,** | **Control +Gefitinib**  vs  **shTERT +Gefitinib** | **Control +Gefitinib**  vs  **shB1 +Gefitinib** | **shTERT +Gefitinib**  vs  **shB1 +Gefitinib** |
| GSH | ns | ns | ns | ns | ns | ns | ns |
| Lac | *p<0.001* | *p<0.05* | *p<0.05* | *p<0.05* | *p<0.01* | *p<0.01* | ns |
| Glc | ns | ns | ns | ns | ns | ns | ns |
| PE | ns | ns | ns | ns | ns | ns | ns |
| Asc | ns | ns | ns | ns | ns | ns | ns |
| Asp | ns | ns | ns | ns | ns | ns | ns |
| Tau | ns | ns | ns | ns | ns | ns | ns |
| GABA | ns | ns | ns | ns | ns | ns | ns |
| Ala | ns | ns | ns | ns | ns | ns | ns |
| Scyllo | ns | ns | ns | ns | ns | ns | ns |
| Cho+GPC+PCh | ns | ns | ns | ns | ns | ns | ns |
| NAA+NAAG | ns | ns | ns | ns | ns | ns | ns |
| mI+Gly | ns | ns | ns | ns | ns | ns | ns |
| Cr+PCr | ns | ns | ns | ns | ns | ns | ns |
| Glu+Gln | ns | ns | ns | ns | ns | ns | ns |

Supplementary Table S2. Quantified lactate concentrations and their corresponding CRLB calculated by LCModel fitting for each group of proton single voxel spectroscopy results.

|  | U251 (TE=16ms) | | GS2 (TE=16ms) | | U251 (TE=144ms) | |
| --- | --- | --- | --- | --- | --- | --- |
|  | Conc | CRLB (%) | Conc | CRLB (%) | Conc | CRLB (%) |
| **shControl +DMSO** | 5.04 ± 1.35 | 7.00 ± 1.55 | 4.20 ± 0.70 | 6.50 ± 1.97 | 4.56 ± 0.55 | 19.67 ± 4.16 |
| **shControl + Gefitinib** | 2.55 ± 0.37 | 5.33 ± 2.66 | 2.20 ± 0.567 | 5.83 ± 1.17 | 2.84 ± 0.47 | 22.67 ± 13.61 |
| **shTERT + DMSO** | 1.17 ± 0.25 | 6.67 ± 1.51 | 1.14 ± 0.44 | 7.83 ± 1.47 | 1.61 ± 0.41 | 26.00 ± 6.56 |
| **shTERT + Gefitinib** | 0.56 ± 0.22 | 10.67 ± 3.33 | 0.51 ± 0.21 | 5.67 ± 1.97 | 0.68 ± 0.39 | 24.33 ± 5.03 |
| **shB1 + DMSO** | 1.02 ± 0.21 | 11.00 ± 3.35 | 1.14 ± 0.303 | 5.33 ± 0.82 | 1.13 ± 0.21 | 17.33 ± 7.09 |
| **shB1 + Gefitinib** | 0.30 ± 0.24 | 10.83 ± 10.94 | 0.30 ± 0.24 | 7.83 ± 4.96 | 0.47 ± 0.35 | 22.00 ± 6.08 |

Supplementary Table S3. Spectral SNR calculated from a non-apodized spectrum by using the maximum height of the largest metabolite signal within 0 to 4.5 ppm area divided by the standard deviation of the noise

|  | U251 (TE=16ms) | GS2 (TE=16ms) | U251 (TE=144ms) |
| --- | --- | --- | --- |
| **shControl +DMSO** | 72.23 ± 20.58 | 62.62 ± 11.43 | 29.96 ± 7.93 |
| **shControl + Gefitinib** | 46.16 ± 8.27 | 65.46 ± 17.32 | 28.61 ± 4.27 |
| **shTERT + DMSO** | 60.88 ± 24.51 | 78.64 ± 19.40 | 29.61 ± 5.06 |
| **shTERT + Gefitinib** | 87.86 ± 21.56 | 94.07 ± 27.20 | 40.30 ± 13.97 |
| **shB1 + DMSO** | 64.56 ± 12.94 | 78.82 ± 19.56 | 33.03 ± 6.06 |
| **shB1 + Gefitinib** | 69.44 ± 17.51 | 86.97 ± 26.22 | 38.34 ± 3.95 |
